# Association of cellular immunity with severity of COVID-19 from the perspective of antigen-specific memory T cell responses and cross-reactivity

**DOI:** 10.1186/s41232-022-00239-1

**Published:** 2022-11-29

**Authors:** Shin-ichiro Fujii, Satoru Yamasaki, Tomonori Iyoda, Kanako Shimizu

**Affiliations:** 1 Laboratory for Immunotherapy, RIKEN Center for Integrative Medical Sciences (IMS), 1-7-22, Suehiro-cho, Tsurumi-ku, Yokohama, 230-0045 Japan; 2 Program for Drug Discovery and Medical Technology Platforms (DMP), RIKEN, Yokohama, Japan

**Keywords:** SARS-CoV-2, Cellular immunity, Adaptive immunity, Memory T cells, Cross-reactivity, Immunotherapy

## Abstract

Coronaviruses regularly cause outbreaks of zoonotic diseases characterized by severe pneumonia. The new coronavirus, severe acute respiratory syndrome coronavirus 2 (SARS-CoV-2), has caused the global pandemic disease COVID-19 that began at the end of 2019 and spread rapidly owing to its infectious nature and rapidly progressing pneumonia. Although the infectivity of SARS-CoV-2 is high, indicated by the worldwide spread of the disease in a very short period, many individuals displayed only subclinical infection, and some of them transmitted the disease to individuals who then developed a severe symptomatic infection. Furthermore, there are differences in the severity of infection across countries, which can be attributed to factors such as the emergence of viral mutations in a short period of time as well as to the immune responses to viral factors. Anti-viral immunity generally consists of neutralizing antibodies that block viral infection and cytotoxic CD8^+^ T cells that eliminate the virus-infected cells. There is compelling evidence for the role of neutralizing antibodies in protective immunity in SARS-CoV-2 infection. However, the role of CD4^+^ and CD8^+^ T cells after the viral entry is complex and warrants a comprehensive discussion. Here, we discuss the protection afforded by cellular immunity against initial infection and development of severe disease. The initial failure of cellular immunity to control the infection worsens the clinical outcomes and functional profiles that inflict tissue damage without effectively eliminating viral reservoirs, while robust T cell responses are associated with mild outcomes. We also discuss persistent long-lasting memory T cell-mediated protection after infection or vaccination, which is rather complicated as it may involve SARS-CoV-2-specific cytotoxic T lymphocytes or cross-reactivity with previously infected seasonal coronaviruses, which are largely related to HLA genotypes. In addition, cross-reactivity with mutant strains is also discussed. Lastly, we discuss appropriate measures to be taken against the disease for immunocompromised patients. In conclusion, we provide evidence and discuss the causal relationship between natural infection- or vaccine-mediated memory T cell immunity and severity of COVID-19. This review is expected to provide a basis to develop strategies for the next generation of T cell-focused vaccines and aid in ending the current pandemic.

## Introduction

Coronaviruses regularly cause outbreaks of zoonotic diseases characterized by severe pneumonia, such as Middle East respiratory syndrome, severe acute respiratory syndrome, and COVID-19. When considering the infection rate and severity of a disease, it is necessary to take into account the factors related to the virus, the host, and the social environment as well as genetic factors. New mutations have recently been reported with the transmission of SARS-CoV-2 between animals (e.g., deer) and humans [1] and may pose a new threat to global health in the future. Antiviral immunity primarily involves neutralizing antibodies and cytotoxic T cells (CTLs). To address simply, neutralizing antibodies can prevent viral infection by recognizing or binding viruses. Since CTLs can recognize the viral epitope on MHC class I of infected cells, CTLs attack and eliminate the infected cells completely. The correlation between immunotypes and clinical outcomes in SARS-CoV-2-infected individuals has been demonstrated. As a component of the progression to severe disease, a dysregulated response involving multiple but linked elements of the host’s immune system was observed in some SARS-CoV-2-infected individuals [2, 3]. As a marker for COVID-19 or the vaccine, the magnitude of the spike-specific antibody response or neutralizing titer has been used in most of the studies. In contrast, much less attention has been paid to the magnitude or functional profile of cellular immunity, because an analysis of cellular immunity particularly for epitope-specific T cells is complex and expensive. However, to understand the mechanisms underlying *SARS-CoV-2* infection, large population cohorts with accurate cellular assays have been recently undertaken. To fully understand the infection outcomes, the relationship between humoral and cellular immunity in the long-term protection against SARS-CoV-2 needs to be investigated. Early studies have discussed that a T cell response can protect from severe infection [4, 5], while others have demonstrated the negative relationship between an active T cell response and severe disease prognosis in patients hospitalized with COVID-19 [6]. Such differences are considered to be due to divergent clinical trajectories as well as disparities in T cell responses and cytokine release syndrome (CRS). In particular, nonspecific immune responses at the onset of COVID-19, such as the production of cytokines like TNF-α and IL-6 from macrophages or IFN-γ from bystander T cells, and lymphopenia have been believed to be risk factors for severity and mortality of COVID-19 [6]. High serum IL-6, IL-8, and TNF-α levels at the time of hospitalization were strong and independent predictors of patient survival [7]. Particularly, TNF-α, one of the pro-inflammatory cytokines commonly upregulated in acute lung injury, triggers CRS and facilitates SARS-CoV-2 interaction with angiotensin-converting enzyme 2 (ACE2) [8]. IL-6 is also the main mediator in patients with COVID-19 with severe respiratory complications [9]. IL-6 level was the most significant predictor of the non-survivors’ group, linking the poor prognosis of these patients to increased IL-6 levels in the context of CRS [10]. A positive correlation between IL-6 and C-reactive protein in acute respiratory distress syndrome was also reported [10].

A recent report using single-cell technologies, such as flow cytometry, mass cytometry, single-cell transcriptomics, and single-cell multi-omics profiling, confirmed the heterogeneity of immune responses among individual cells and COVID-19 pathogenesis [11]. In severe COVID-19, several characteristic immune cell subsets that secrete inflammatory cytokines were increased. The frequency of NKG2C^+^CD57^+^CD56^dim^NK cells and CD56^high^NK cells, S100A8 or S100A9^high^ neutrophils, HLA-DR^low^ monocytes, and FCN1 (a member of the complement cascade) or SPP1 (a pro-inflammatory cytokine)-expressing macrophages was increased and related to the severity [11]. In contrast, not only CD4^+^ and CD8^+^ T cells but also the CD8/CD4 ratios were remarkably decreased in severe COVID-19 cases. In addition, the COVID-19-related CRS in multisystem inflammatory syndrome (MIS-C) in children is characterized by IFN-γ as a crucial cytokine in the communication among HLA-DR^+^/TIM3^+^/CD38^+^T cells, patrolling monocytes, and CD16^+^ NK cells [12]. Another group reported that the abundance of blood innate lymphoid cells (ILCs) correlated inversely with the severity of the disease in SARS-CoV-2-infected adults, and these blood ILCs resembled lung ILC2 capable of producing amphiregulin to promote tissue protection [13].

Given that the immune system has not encountered SARS-CoV-2 before 2019, it lacks a reliable historical record to control the virus. When some patients who had been exposed to SARS-CoV-2 and experienced an overexuberant innate or adaptive immune response, an impaired immune regulation with immunopathology could often entail overcompensation from the arms of the immune system. Meanwhile, an insufficient T cell immunity did not respond to viruses well because of a sub-optimal control over the invading pathogen. This review comprehensively discusses the adaptive immunity and COVID-19 severity by focusing on CD4^+^ and CD8^+^ T cell immunity.

### Relation between cellular immunity and COVID-19 disease phenotype

After the pandemic hit, several studies carefully reported the SARS-CoV-2-specific T cell response in terms of phenotypic and functional properties. After the onset of symptoms, the immune responses in the acute and memory phases (6 months post-infection) were assessed. The virus-specific T cell responses were reported to show a half-life of approximately 3–5 months [14–21]. The relationship between the T cell response and disease severity should be assessed at different time points post-infection. Indeed, when the initial failure to control the infection does not drives the T cell response correctly but elicits functional profiles that inflict tissue damage without effectively eliminating viral reservoirs, a vicious cycle occurs in the early phase of infection. In an early study, SARS-CoV-2-specific CD4^+^ and CD8^+^ T cells and neutralizing antibody responses in 28 acute and 15 convalescent subjects were analyzed [14]. SARS-CoV-2-specific T cell responses were associated with a milder disease, but not neutralizing antibodies, indicating roles for both CD4^+^ and CD8^+^ T cells in protective immunity against COVID-19. The CD4^+^ T cell response is induced early during SARS-CoV-2 infection, but the CD8^+^ T cell response takes more time to build up post-infection [22]. CD8^+^ T cells in non-symptomatic outpatients were found to increase more rapidly than that in patients with severe infection [23]. When blood samples from 37 patients who were hospitalized in the acute phase of COVID-19 were analyzed around 2 weeks after symptoms onset, the number of both spike (S) protein- and nucleocapsid (N)-specific CD4^+^ T cells with polyfunctionality (IL-2^+^TNF-α^+^ and IL-2^+^IFN-γ^+^) in patients with mild disease was higher than that in patients with severe infection. S-specific polyfunctional CD8^+^ T cells were also higher in patients with mild disease than in those with severe disease [24], whereas there is an apparent difference in the expression of coinhibitory molecules between patients with mild and severe diseases. S-specific CD8^+^ and CD4^+^ T cells in patients with severe disease express PD-1, TIM-3, CD39, VISTA, and Galectin-9 compared to those in patients with mild disease [25]. Thus, virus-specific T cell response was different between patients with mild and severe diseases.

Differences in T cell responses in children and older individuals have also been described. Infected children mostly follow a milder disease course. Significantly reduced CD4^+^ and CD8^+^ T cell responses were observed in children with mild COVID-19 compared with those in adults [22]. In contrast, it should be noted that enhanced T cell responses have also been reported in pediatric patients [26]. Since these data were obtained by a short-term assay, whether low immune responses were caused due to low frequency or compromised function of T cells cannot be determined. Interestingly, there is no difference in the frequency of antigen-specific CD8^+^ T cells between asymptomatic and symptomatic children [27], nor between MIS-C and convalescent pediatric patients [28]. Despite the relatively lower cellular responses to ORF peptide or N- and membrane (M)-derived peptides, a two-fold greater T cell response was detected against S-derived peptides in children [26]. Thus, at least S protein-responsive T cell function is preserved in children. Several reports also showed other immunological characteristics of T cells in children: they may harbor a greater stem cell memory (TSCM) subset of memory T cells [29]. Increased tissue-resident T (TRM) cell subsets of SARS-CoV-2 reactive pre-existing CD8^+^ T cells have also been observed in the tonsils of children [30]. Such evidence supports the notion that SARS-CoV-2-specific CTL or memory T cells are quickly and robustly activated in children following exposure to the SARS-CoV-2 antigen. This may be due to children catching a common cold frequently. In contrast, individuals who are more than 65 years old display a propensity to develop severe disease outcomes, because of aging-related impairments in the immune-regulatory mechanisms [14, 31, 32]. Particularly, the cytotoxic T cell potential, manifested as granzyme and perforin in effector memory and terminally differentiated effector CD8^+^ T cells, was diminished or impaired in elderly people over the age of 80 years, making severe COVID-19 more likely in these patients [33]. These findings are consistent with previous reports of age and gender-related differences in the potency of lymphocyte responses.

The distribution and subsets of SARS-CoV-2-reactive T cells in COVID-19 patients have been demonstrated. T cell subsets are generally defined based on CD45RA and CCR7 or CD27 and CD95, naïve T (Tnaive) (CD45RA^+^CCR7^+^ or CD45RA^+^CD27^+^CD95^−^), stem cell memory (TSCM) (CD45RA^+^CCR7^+^ or CD45RA^+^CD27^+^CD95^+^), central memory (TCM; CD45RA^−^CCR7^+^ or CD45RA^−^CD27^+^), effector memory (TEM; CD45RA^−^CCR7^−^ or CD45RA^−^CD27^−^), terminally differentiated effector T (TEMRA; CD45RA^+^CCR7^−^ or CD45RA^+^CD27^−^), and tissue-resident memory T (TRM; CD45RA^−^CCR7^−^ or CD45RA^−^CD27^−^CD69^+^ and/or CD103^+^) [34]. SARS-CoV-2-specific T cell subsets of CD4^+^ and CD8^+^ TEM cells and CD8^+^ TEMRA cells have been detected primarily in the bone marrow (BM), spleen, and gut-associated lymph nodes (LN), in addition to the lungs and lung-draining LN [35], whereas the canonical CD4^+^ and CD8^+^ TRM cells have been dominantly observed in the lungs of convalescences [35]. Some of the memory T cells specific for the S, M, and N regions were maintained for at least 10 months or 1 year after infection regardless of disease severity [36, 37]. HLA-peptide-multimer analysis has shown that SARS-CoV-2-specific CD8^+^ T cells are maintained as TCM or TSCM in the blood of recovered individuals and simultaneously can differentiate into TEM and TEMRA upon re-exposure to the antigens [36, 38]. As several studies implicate T cells to be protective based on the associations with symptoms and outcomes, the characterization of T cells is crucial.

### SARS-CoV-2-specific T cells in relation to HLAs in COVID-19 disease phenotype

Immunodominant epitopes from structural proteins, such as S, M, and N proteins, or non-structural proteins (NSPs), such as NSP7 and NSP13 encoded on ORF1 of SARS-CoV-2, have been predicted and detected in infected patients and convalescent individuals [15, 16, 19, 20, 39–42]. A virus-specific CD4^+^ and CD8^+^ T cell response against S, M, and N proteins was predominantly noted. CD4^+^ T cells responded to NSPs and ORF8, whereas CD8^+^ T cells targeted nsp6, ORF8, and ORF3a; these comprised 32% of the total CD8^+^ T cell pool [15].

**Fig. 1:**
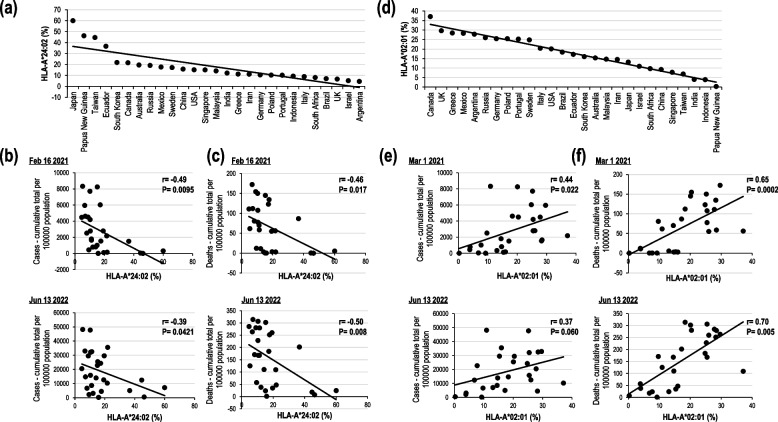
Data on infection and death caused by SARS-CoV-2 in the HLA-A*02:01- or HLA-A*24:02-positive population worldwide at two time points. (**a**) Data on the HLA-A*24:02-positive population in the indicated countries, which is derived from the Allelic Frequency Net Database (AFND, http://www.allelefrequencies.net/) [54]. (**b**, **c** )Correlation between HLA- A*24:02 and (**b**) COVID-19 cases [cumulative total per 10^5^ population] and (**c**) COVID-19 deaths [cumulative total per 10^5^ population]. These data were derived from the WHO Coronavirus (COVID-19) Dashboard (https://covid19.who.int/). (**d**) Data on the HLA-A*02:01-positive population in the indicated countries. (**e**, **f**) Correlation of HLA-A*02:01 with (**e**) COVID-19 cases [cumulative total per 10^5^ population] and (**f**) COVID-19 deaths [cumulative total per 10^5^ population]. Pearson’s correlation coefficients (Pearson’s *r*) are shown

Although sequence homology data for SARS-CoV-2 with other human CoVs and state-of-the art bioinformatic approaches may lead us to the candidate SARS-CoV-2 antigens, HLA restriction for virus-specific T cells should be considered. With respect to HLA restriction in CD4^+^ and CD8^+^ T cells, an in-depth analysis of HLA-restricted SARS-CoV-2-specific T cell responses has been reported by focusing on several HLAs (Table 1). As an in silico method does not clarify whether they actually respond or not, we corroborated the results with immunological data. To understand the relationship between HLA alleles and COVID-19 in terms of the immune response against SARS-CoV-2, we initially evaluate the frequency of two prominent HLA populations in various countries, and then compared the relation between infection rate and number of COVID-19-related deaths at two different time points—before and after global vaccination (early 2021 and 2022)—in populations with prominent HLA class I alleles, HLA-A*02:01 and A*24:02 (Fig. 1). HLA-A*24:02 is distributed in ~ 60% of the Japanese population [54]. Other populations in which HLA-A*24:02 is present in a high percentage of the investigated members are various indigenous populations in Asia, Oceania, and the Americas. HLA-A*02:01 is common in Europe and, for example, found in 49% of the Polish population (based on AFND) [54]. Although there may also be several other reasons, such as social and medical differences for the infection rate and mortality, there was an inverse correlation between the COVID-19-related deaths per 100,000 population and the HLA-A*24:02^+^ population in the indicated countries (Fig. 1a, c), which did not hold true for the HLA-A*02:01^+^ population (Fig. 1d, f). Since the evidence of the inverse relation between HLA class I and COVID-19 severity may possibly be reflected via the difference of CD8^+^ T cell response, we initiated the immunological analysis of the HLA class I-restricted CD8^+^ T cell response.

**Table 1:** Immunodominant peptides and associated HLA class I

Protein	Start	End	Length	Peptide	HLA Class I	Source
S	865	873	9	LTDEMIAQY	A^*^01:01	ref. [42], ref. [43]
M	171	179	9	ATSRTLSYY	A^*^01:01	ref. [44], ref. [43]
nsp3	819	828	10	TTDPSFLGRY	A^*^01:01	ref. [40], ref. [44], ref. [45]
nsp3	503	511	9	PTDNYITTY	A^*^01:01	ref. [44], ref. [43]
nsp3	818	828	11	HTTDPSFLGRY	A^*^01:01	ref. [46], ref. [45]
nsp5	174	182	9	GTDLEGNFY	A^*^01:01	ref. [44], ref. [43]
nsp12	738	746	9	DTDFVNEFY	A^*^01:01	ref. [44], ref. [42], ref. [43]
ORF3a	207	215	9	FTSDYYQLY	A^*^01:01	ref. [44], ref. [41], ref. [42], ref. [45]
S	269	277	9	YLQPRTFLL	A^*^02:01	ref. [44], ref. [38], ref. [47], ref. [45], ref. [43]
S	1000	1008	9	RLQSLQTYV	A^*^02:01	ref. [47], ref. [45], ref. [43]
S	424	433	10	KLPDDFTGCV	A^*^02:01	ref. [43], ref. [48]
nsp3	1514	1522	9	ILFTRFFYV	A^*^02:01	ref. [42], ref. [45], ref. [43]
nsp4	337	344	8	FLPGVYSV	A^*^02:01	ref. [45], ref. [43]
nsp7	27	35	9	KLWAQCVQL	A^*^02:01	ref. [44], ref. [43]
nsp8	152	160	9	ALWEIQQVV	A^*^02:01	ref. [44], ref. [43]
ORF3a	139	147	9	LLYDANYFL	A^*^02:01	ref. [44], ref. [38], ref. [43]
ORF3a	107	115	9	YLYALVYFL	A^*^02:01	ref. [42], ref. [43]
N	361	369	9	KTFPPTEPK	A^*^03:01 A^*^11:01	ref. [44], ref. [41], ref. [45]
N	134	143	10	ATEGALNTPK	A^*^11:01	ref. [40, 49], ref. [44], ref. [45]
S	448	456	9	NYNYLYRLF	A^*^24:02	ref. [50], ref. [45], ref. [43]
S	1208	1216	9	QYIKWPWYI	A^*^24:02	ref. [51], ref. [40], ref. [44], ref. [45], re [43].
nsp3	1349	1357	9	NYMPYFFTL	A^*^24:02	ref. [45], ref. [43]
nsp13	397	405	9	VYIGDPAQL	A^*^24:02	ref. [40], ref. [44]
ORF3a	112	120	9	VYFLQSINF	A^*^24:02	ref. [40], ref. [44], ref. [43]
N	105	113	9	SPRWYFYYL	B^*^07:02	ref. [44], ref. [41], ref. [42], ref. [45], ref. [43], ref. [52]
N	257	265	9	KPRQKRTAT	B^*^07:02	ref. [42], ref. [45]
nsp13	592	600	9	IPRRNVATL	B^*^07:02	ref. [44], ref. [42]
S	919	927	9	NQKLIANQF	B^*^15:01	ref. [38]
N	9	17	9	QRNAPRITF	B^*^27:05	ref. [40], ref. [41]
N	322	331	10	MEVTPSGTWL	B^*^40:01	ref. [40], ref. [41], ref. [42], ref. [45], ref. [53]

Table 1 lists the several specific HLA-restricted peptides that have been reported to date as well as CD8^+^ T cell responses that have been confirmed in more than two studies. First, the biological T cell response in HLA-A*24:02 was assessed [51]. The potent high CTL precursor frequency for S_1208–1216_ (QYIKWPWYI; QYI) peptide, which is in the S region, could be detected in peripheral blood mononuclear cells (PBMC) of HLA-A*24:02-unexposed healthy donors (UHDs) in early 2020 or pre-pandemic era (2004~2010). S_448–456_ (NYNYLYRLF; NYNY) peptide under HLA-A*24:02 has also been reported as the immunodominant epitope [50]. According to the restriction of the HLA-A*02:01 allele, SARS-CoV-2 epitopes, e.g., S_269–277_ (YLQPRTFLL; YLQ) and S_1000–1008_ (RLQSLQTYV; RLQ) peptides, were identified to be most responsible for the CD8^+^ T cell response [43, 47, 50, 55]. However, the precursor T cells for YLQ seem low [56]. Also, YLQ-specific CD8^+^ T cell response in acute and convalescent patients did not involve the expression of T cell activation markers, such as CD38, HLA-DR, PD-1, and CD71 [57], suggesting an insufficient and tepid response. Similar to HLA-A*02:01 allele, because the HLA-B*07:02 in the human population is also major, several groups have tracked SARS-CoV-2-specific CD8^+^ T cell responses restricted to this allele in conjunction with the clinical course. The CD8^+^ T cells in HLA-B*07:02 UHDs and patients respond well to the nucleoprotein epitope (N_105–113_; SPR peptide). The SPR peptide-B*07:02-specific CD8^+^ T cell responses are considered among the most dominant in SARS-CoV-2-infected individuals [58]. In their study, the SPR peptide-specific T cell response has been associated with mild disease. In addition, the specific T cell clones showed not only high functional avidity, but also antiviral efficacy. An immunodominant T cell response against residues 322 to 331 of nucleocapsid (Nuc_322–331_; MEVTPSGTWL; MEV peptide) was assessed using HLA-B*40:01/MEV tetramer, which is conserved in all variants of concern (VOCs) reported to date, but is not cross-reactive to seasonal coronaviruses [53]. Over a 6-month period of convalescence that was monitored, MEV peptide-specific CD8^+^ T cells showed the TSCM phenotype and revealed the polyfunctional capacity. These epitope-specific CTLs can not only be expanded in case of SARS-CoV-2 infection, but also highly detected in convalescents, indicating that these would be protective. These epitope-specific CTLs can be expanded in some subjects, but not in others by the current mRNA vaccine [59, 60], which requires further investigation. Thus, immunodominant epitopes restricted in a specific HLA manner were identified and analyzed for CD8^+^ T cell responses. Tracking of SARS-CoV-2-specific CD8^+^ T cell responses restricted to these alleles in conjunction with the clinical course will yield a wealth of valuable immunological data relevant to the pandemic.

### Characterization of SARS-CoV-2-specific memory T cells in COVID-19 disease

There are two types of anti-viral CTLs: one is specific against only SARS-CoV-2, and the other is cross-reactive CTL, which is reactive to SARS-CoV-2 as well as other HCoVs*.* The pre-existing immunity could influence the severity of disease associated with subsequent SARS-CoV-2 infection and/or the outcomes of SARS-CoV-2 vaccination [61]. In fact, it was reported that recent exposure to seasonal coronaviruses correlated with less severe COVID-19 outcomes [62]. Although such a cross-reactivity with pre-existing T cells has been reported [15, 16, 18–20, 40, 61], whether these pre-existing cross-reactive T cells play a protective or pathological role in the hitherto uninfected host when exposed to SARS-CoV-2 was initially unknown [63]. Two studies showed that SARS-CoV-2 cross-reactive CD4^+^ T cells may exhibit low avidity and be non-protective, i.e., antigenic sin [64, 65]. The concept of “antigenic sin,” a term derived from antibody responses, indicates an altered immune response upon rechallenge with a closely related antigen. Once antigen-specific T cells with low affinity are established in individuals previously exposed an antigen, weak or no T cell responses would be sometimes observed upon rechallenge with the closely related or mimicking antigen. Therefore, the immunodominant epitope should be identified in order to generate cross-reactive T cells with high affinity. In fact, other recent papers show that cross-reactive T cells play a protective role [52, 58, 66, 67]. Overall, cross-reactive memory CD4^+^ T cells recognizing SARS-CoV-2 were detected in approximately 50% of individuals pre-pandemic and found to have functional properties against COVID-19. Similar evidence has been reported by studies wherein seronegative healthcare workers had high levels of seasonal coronaviruses-T cell reactivity [68], and cross-reactive T cells were associated with abortive SARS-CoV-2 infection in healthcare workers [69]. The levels of cross-reactive CD8^+^ T cells in convalescent individuals who had experienced mild disease were much higher than those in infected patients who had experienced severe disease [70]. Such a cross-reactivity will be discussed below in the “SARS-CoV-2 cross-reaction of memory T cells with seasonal coronaviruses” section. In addition, from the point of view of cross-reactivity to seasonal coronavirus, T cell cross-reactivity to SARS-CoV-2 variants should be expected; this is further discussed in the “T cell cross-reactive response against variants of SARS-CoV-2” section.

#### SARS-CoV-2 cross-reaction of memory T cells with seasonal coronaviruses

**Fig. 2:**
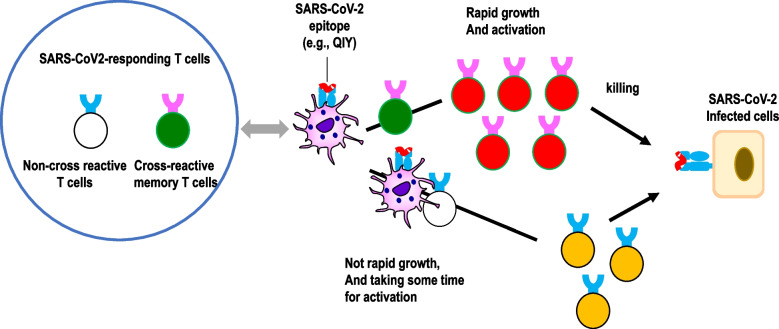
Different roles of specific and cross-reactive T cells in the immune response to SARS-CoV-2 infection. When an optimal epitope with high activation potential is identified, cross-reactive T cells proliferate rapidly and robustly in a short period of time, resulting in a strong anti-viral response. In case of specific, non-cross-reactive T cells against SARS-CoV-2, T cell activation requires dendritic cells for priming, which takes time (approximately 1–2 weeks for activation and over a month for memory T cell induction)

Certain strains of HCoV (HKU1, OC43, 229E, and NL63) are often endemic in human populations and cause ~ 20% of upper respiratory tract infections. Furthermore, the potential exposure to animal β-coronaviruses for cats or dogs might affect the appearance of coronavirus variants [71, 72]. In 2020, it remained unclear whether pre-existing HCoV-specific CD8^+^ T cells were converted to functionally competent T cells that cross-react with SARS-CoV-2. The difficulty in verifying the cross-reactivity of the T cells against HCoV lies in the fact that both structural and non-structural proteins have been reported as epitopes recognized by cross-reactive T cells, owing to which a very large number of epitopes must be examined. Although such T cell cross-reactivity is a useful system to maximize the use of the limited number of CTLs against various mutated SARS-CoV-2 (Fig. 2), it is important to determine whether it actually functions in a protective manner against pathogens, or whether the increase in non-protective T cell clones may lead to the original antigenic sin phenomenon. Although there is usually a specific combination between HLA and T cells in the presence of antigen, T cell cross-reactivity can be defined as the recognition of more than one distinct peptide-MHC structure by a single T cell receptor (TCR). For a detailed study of T cell cross-reactivity, it is necessary to determine a specific HLA to examine.

As mentioned above, HLA-A*24:02 is more common in some countries including the Japanese population and is inversely related to the severity of disease (Fig. 1). We therefore studied the relation between HLA-A*24:02 and CTLs. We selected epitopes of the SARS-CoV-2 S protein with high affinity to HLA-A*24:02 based on in silico analysis and screened them in an in vitro study using HLA-A*24:02-positive donor samples. We identified an HLA-A*24:02-high binding, immunodominant epitope (QYI peptide) in the SARS-CoV-2 spike region that can be recognized by seasonal coronavirus-specific CD8^+^ T cells from more than 80% of HLA-A*24:02^+^ UHDs [51]. Six out of nine amino acids were conserved between the QYI peptide and the relevant peptides derived from seasonal coronavirus. CD8^+^ T cells in response to the selected dominant epitope (QYI peptide) displayed multifunctionality and cross-functionality across HCoVs and SARS-CoV-2 in HLA-A*24:02^+^ donors. Since the proof of the cross-reactivity needs to be proved at a single cell level, the cross-reactivity and functional avidity of QYI-specific TCRs were confirmed at the single TCR level. Furthermore, we identified hot spot epitope areas as immunodominant epitopes for HLA-A*24:02, including QYI peptide, which were covered by three 15-mer-long peptides [51].

In addition, there are several recent studies. Schulien et al. performed an analysis of pre-existing and induced CD8^+^ T cells in three patients with mild disease pre- and post-SARS-CoV-2 infection using peptide-loaded major histocompatibility complex class I (pMHCI) tetramer strategy and found rapid induction, prolonged contraction, and emergence of heterogenous and functionally competent cross-reactive induced memory CD8^+^ T cell response [42]. Mallajosyula et al. demonstrated that T cells that recognize peptides conserved among coronaviruses are abundant in UHDs. They also detected shared TCR motifs of SARS-CoV-2 specific CD8^+^ T cells between UHDs and patients with mild COVID-19 [70], suggesting a protective role of pre-existing CD8^+^ T cells in COVID-19. Lineburg et al. showed the presence of immunodominant, HLA-B7-restricted SARS-CoV-2 nucleocapsid protein epitope (SPR peptide) cross-reactive CD8^+^ T cells [52]. Specifically, they showed the presence of a shared CDR3β motif in epitope-specific CD8^+^ T clonotypes in exposed and unexposed donors, which suggested that pre-existing immunity in HLA-B7^+^ individuals favored clonal expansion. Taken together, both QYI and SPR peptides are known as HLA-A*24:02 and HLA-B*07:02-restricted cross-reactive peptides to HCoVs [51, 52, 58]. This indicates that some of UHDs bearing relevant HLAs have pre-existing CTLs. Thus, it is necessary to identify the immunodominant epitope and ensure whether the pre-existing CD8^+^ T cells with high avidity cross-react against SARS-CoV-2.

Meanwhile, the SPR-specific CD8^+^ T cell response differs in patients with mild disease and severe disease after infection with SARS-CoV-2 [58]. The study found that NP_105–113_-B*07:02 is the dominant NP response in HLA-B*07:02-positive patients with mild symptoms, with high frequency and higher magnitude when compared with that in severe cases [58]. In addition to memory fraction, some SPR-specific CD8^+^ T cells originate from a naïve fraction [38, 58]. However, further studies are required to ascertain the functional status of the cross-reactive T cells and examine whether they can respond rapidly.

To identify cross-reactive CD4^+^ T cell responses, *≥* 67% amino-acid sequence homology in epitope is believed to be a reliable cutoff for predicting CD4^+^ T cell cross-reactivity [16]. Approximately 20–50% of UHDs display cross-reactive CD4^+^ T cell responses to the SARS-CoV-2 S, M, and N proteins, as well as NSPs (nsp4, 6, 7, 13 and 14) [15–20]. Loyal et al. reported that the fusion peptide domain of spike protein (S_816–830_) was a universal immunodominant epitope for CD4^+^ T cells and recognized by CD4^+^ T cells in 20% of UHDs, 50–60% of COVID-19 convalescents, and 97% of mRNA vaccinated subjects [67]. It suggests that these cross-reactive CD4^+^ T cells could be rapidly recruited into the immune response following SARS-CoV-2 infection or mRNA vaccination.

Both the high functional avidity and polyfunctionality of cross-reactive T cells will teach us the relation to the protective potential. The extent to which antibody titers and the degree to which SARS-CoV-2-specifc CD8^+^ T cells are detected are related to the disease severity needs to be verified. Carefully designed prospective studies evaluating the clinical outcomes of SARS-CoV-2 infection will be further necessary to provide. At least, several types of cross-reactive CD8^+^ T cells and type 1 helper T cell (Th1) CD4^+^ T cells that support CTL functions [67, 73] can influence disease severity and outcome [74, 75]. The importance of cross-reactive, CD4^+^ follicular helper T (Tfh) cells that support antibody production remains to be determined [76, 77].

#### T cell cross-reactive response against variants of SARS-CoV-2

The abovementioned observations also imply the possibility of cross-reaction with variants of SARS-CoV-2. SARS-CoV-2 has mutated to adapt to its host as a result of rigid selection pressure by adaptive immunity. As a consequence, new variants have emerged, some of which are classified as VOCs in that these variants may show greater transmissibility and/or are better able to escape immunity [78]. Therefore, they may often cause higher mortality than the original strain that originated in Wuhan.

T cell response to variants of SARS-CoV-2 is different from the T cell cross-reactivity between HCoVs and SARS-CoV-2. Therefore whether T cells responding to SARS-CoV-2 can also protect against variants needs to be examined. With regard to the adaptive immune responses against SARS-CoV-2 variants in COVID-19 convalescents [45], 93% and 97% of previously identified CD4^+^ and CD8^+^ T cell epitopes (i.e., B.1.1.7, B.1.351, P.1, and CAL.20C lineages) were not affected by mutations [45]. The decrease in T cell response against SARS-CoV-2 variants compared to Wuhan was small, suggesting that it is important to monitor active T cell reactivity in the context of SARS-CoV-2 evolution. In another study, by examining the ability of T cells to react to Omicron S protein in participants who were vaccinated or unvaccinated convalescent patients (*n* = 70), approximately 70% and 80% of the CD4^+^ and CD8^+^ T cell response to the S protein derived from the strain that originated in Wuhan was maintained [79]. The magnitude of the Omicron cross-reactive T cells was similar to that for the Beta (B.1.351) and Delta (B.1.617.2) variants. There were also comparable T cell responses in Omicron-infected patients to those hospitalized in previous waves with the ancestral, Beta, or Delta variants (*n* = 49). This study also indicates that T cell response against variants was mostly preserved. Therefore, these two reports highlight the importance of identifying key immunodominant epitopes in the mutated virus as well as analysis of antigen-specific T cell responses. In fact, most of the epitopes (YLQ, RLQ, and QYI) identified above have been conserved among the SARS-CoV-2 variants. However, the NYNY epitope was mutated in case of the Delta, Epsilon, and Kappa variants, and NYNY-specific CTLs cannot recognize the mutated epitope [50]. HLA-A*01:01-restricted nsp3 (TTDPSFLGRY), ORF3a (FTSDYYQLY), HLA-A*03:01/A*11:01-restricted nucleocapsid (KTFPPTEPK), HLA-B*27:05-restricted nucleocapsid (QRNAPRITF), and HLA-B*40:01-restricted nucleocapsid (MEVTPSGTWL) were also reported to show partial or complete loss of CD8^+^ T cell responses for their variant epitopes [80]. Regarding the SPR peptide, mutation was reported in the delta strain, and a loss of recognition by SPR-specific CTLs was predicted because of the decrease of HLA epitope binding [81]. Despite extensive mutations and reduced susceptibility to neutralizing antibodies of Omicron, the majority of T cell responses induced by vaccination or infection cross-reacted with the variants. If cross-reactive epitopes were selected optimally in terms of CTL induction, the epitope can elicit CTLs against variants, even if they have any other mutated sites that are no longer recognized by antibodies. Nonetheless, the identified mutations are of concern, and it would be worthwhile to identify key immunodominant epitopes in the mutated virus.

### T cell response in COVID-19 vaccine recipients

Vaccine production against viruses is generally developed with the main target of antibody induction, but the concurrent immune response differs with the formulation of each vaccine. Previous studies demonstrated that inactivated influenza viral vaccines do not induce CD8^+^ T cell immunity, but Tfh and/or antibody production [82–85]. SARS-CoV-2 vaccine has been focused on the development of targeting the critical S protein for viral entry and has been produced using mRNA and viral vector systems. Particularly, SARS-CoV-2 S-targeting vaccines by vaccine platforms (mRNA-1273, BNT162b2, Ad26.COV2.S, and NVX-CoV2373), mRNA lipoparticles (Moderna VRC mRNA-1273 [86] and Pfizer BNT162b2 [87]), Ad26.COV2.S, and NVX-CoV2373 have been used globally.

T cell response elicited by the mRNA vaccine has been analyzed by a study using peptide pools in which vaccination was shown to induce rapid antigen-specific CD4^+^ T cell responses in naive subjects after the first dose, whereas CD8^+^ T cell responses were found to develop gradually and were variable in magnitude [88]. Furthermore, vaccine-induced CD4^+^Th1 cells and circulating CD4^+^Tfh cell responses after the first dose correlated with post-boost CD8^+^ T cells and neutralizing antibodies, respectively [88]. Although these vaccines were biased toward the expression of Th1 cytokines, minimal Th2 cytokine expression (IL-4 and IL-13) and after a while CD8^+^ T cell responses to the S protein were elicited at low level (< 0.1% in blood CD8^+^ T cells; Moderna) and a certain level (0.02–2.92% in blood CD8^+^ T cells; BioNTech) even after the second vaccination [59, 89, 90]. These findings suggest that the CD8^+^ T cell response depends on the establishment of CD4^+^ Th1 T cells. In a study tracking CD8^+^ T cell response to immunodominant antigen peptide using tetramer, it was reported that antigen-specific CD8^+^ T cells can be expanded much earlier (peak on days 9–12 after the first dose) [59, 89, 90]. The expansion of CD8^+^ T cells may partly depend on the pre-existing T cells (Fig. 2).

The next important issue for evaluating vaccines is to determine how long the T cell responsivity persists after vaccination, memorization, and reactivity to mutant strains. In the case of the mRNA vaccine, CD4^+^ and CD8^+^ T responses last as long as in the case of SARS-CoV-2 infection. When analyzing the response using peptide pool assays, CD4^+^ and CD8^+^ T cells are detected only for 3–6 months in the peripheral blood, post which the levels return to pre-vaccine levels [59, 91]. Most SARS-CoV-2 S-specific memory CD8^+^ and CD4^+^ T cells driven by COVID-19 vaccination recognize and respond to the S protein from the variants including Omicron [79, 92]. These reports indicate that SARS-CoV-2 S-specific memory CD8^+^ and CD4^+^ T cells are elicited, or preexisting T cells are enhanced by COVID-19 vaccination. Indeed, although many viral VOCs can strongly evade humoral immunity, T cell responses induced by vaccines show strong cross-protection against VOCs and support the concept that cellular responses contribute substantially to disease control. Especially, regarding the effect of vaccines in immunocompromised individuals, for example, patients with cancer, it was reported that the specific T cell response was detectable even when the production of neutralizing antibodies was impaired [93]. In a recent article on risk analysis of breakthrough infections (December 1, 2020~May 31, 2021), the risk is still high in patients with cancer, especially in those with hematopoietic tumors (adjusted odds ratios (OR) ranged from 2.07 for lymphoma to 7.25 for lymphoid leukemia) and that patients with multiple myeloma have a higher risk of severe outcomes (adjusted OR 1.75) [94]. Another report also demonstrated that immunocompromised people have a higher risk (13.6%) of breakthrough infections compared to the non-immunocompromised population (4.9%) [95]. For this purpose, it is important to know whether measuring T cell responses adds to the predictive ability of our existing B cell immune correlates. Thus, a more optimized T cell-based vaccine may be needed for immunocompromised patients.

### Development of a new type of T cell targeting vaccine

T cell responses in severely ill individuals show evidence of T cell decrease, T cell dysfunction, or T cell tolerance, suggesting that a failure of T cell reactivity to elicit productive control may drive them toward pathological states that contribute to disease propagation. T cell-targeting vaccines have the potential to generate effective T cells against SARS-CoV-2 rather than antibodies, and such studies have been conducted in animals and humans [49, 96, 97]. In these animal models of high-dose infection, high levels of vaccine-induced CD8^+^ T cells (approximately 4.5% of total CD8^+^ T cells) in the lung reduced the viral load and protected the animals from the disease [98]. The frequency of CD8^+^T cells would be the index value for developing the T cell vaccine. There are two approaches for T cell-targeting vaccine development. The first one employs peptides to expand pre-existing memory CD8^+^ T cells. As discussed in the cross-reactivity session, the QYI peptide as well as immunodominant epitopes composed of three long peptides involving QYI would be candidates for T cell-based peptide vaccines [51]. In fact, the long peptide including the QYI peptide can induce SARS-CoV-2-targeting CTLs in 100% of HLA-A24^+^ healthy subjects and 70% of patients with hematologic cancers. Several other peptide-based vaccines have also shown potential [49, 97, 99]. Clinical trials of T cell-targeted peptide vaccines for individuals with high-risk B cell tumors are currently ongoing (ClinicalTrials.gov Identifier: NCT05113862, NCT04954469) [49]. The second approach is through the utilization of DCs in situ in order to prime naïve T cells into antigen-specific CTLs. This strategy can induce higher levels of CTLs. We previously reported the development of artificial adjuvant vector cells for SARS-CoV-2 (aAVC-CoV-2) as an in vivo DC-targeting approach using SARS-CoV-2 mRNA-transduced cells and demonstrated long-term memory T cell induction [96]. CTL induction can be compared between mRNA vaccines and aAVC-CoV-2. A preclinical study of mRNA vaccines demonstrated that SARS-CoV-2 S-antigen-specific IFN-γ-producing CD8^+^ T cells at the effector phase amounted to approximately 4.5% of total CD8^+^ T cells in the lungs of mice vaccinated with 30 μg mRNA/mouse [98]. We reported that S-antigen-specific IFN-γ-producing CD8^+^ T cells were 25% of total CD8^+^ T cells in the lungs of mice vaccinated with aAVC-CoV-2 (3 μg of CoV-2-S mRNA). By comparing the results of these two studies, vaccination with aAVC-CoV-2, harboring a 10-fold lower amount of CoV-2-S mRNA, resulted in the generation of up to 10-fold higher levels of antigen-specific CD8^+^ T cells relative to the currently used mRNA vaccine. Moreover, aAVC-CoV-2 induced higher numbers of long-term memory CTLs [96]. In a phase I trial against cancer, we have just reported the induction of innate and adaptive immunity in addition to safety in refractory and relapse AML patients using a WT1 mRNA-expressing aAVC [100]. It could be developed for viral protection in humans. From the evidence, the aAVC system would also have a potential for the generation of CTLs against SARS-CoV-2 in humans.

## Conclusion

A role for T cells has been reported as being protective based on associations with symptoms and outcomes and is predicted to protect from progression to severe SARS-CoV-2 and breakthrough infection. We monitored the infection and disease severity rates at two time points in 2021 and 2022 (Fig. 1). Considering the increase in SARS-CoV-2 infection rate, although with limited severity in Japan (July to August 2022), it is worthwhile to understand not only the function of neutralizing antibodies, but also the T cell response against SARS-CoV-2. The extent to which CD4^+^ and CD8^+^ T cells provide protection and limit disease severity of SARS-CoV-2 infection in humans has not been rigorously defined. This is because determining the role of T cells alone requires the study of the magnitude and timing of recall responses in individuals with breakthrough infections, and also, the investigation of how the level of CD4^+^ and CD8^+^ T cells predicts either viral clearance or the severity of the clinical outcome. Current SARS-CoV-2 vaccines have benefited the majority of healthy people but result in relatively low levels of circulating CD8^+^ T cells (0.1% of total CD8^+^ T cells) and CD4^+^ T cell responses in humans. This suggests that the current levels of response seen in humans may be much lower than that observed in mice.

Studies of breakthrough infection in immunocompromised and immunosuppressed individuals may be important. Since impaired cellular immunity may contribute to a poor outcome even after vaccination, immunocompromised patients have a higher risk of breakthrough infections. Particularly, in patients with defective or absent B cell compartment but a functional T cell compartment, the T cell response may be perturbed. Determining the type of immune responses that can contribute to protection is clearly a major priority in the highly vulnerable population. Insufficient immunogenicity of vaccines also should be a hallmark of susceptibility to infection for high-risk patients. To find approaches not only to induce virus-specific T cell responses but also T cell boosting could provide a novel avenue for the protection of the at-risk groups. Further studies are needed to provide evidence to support the ongoing study and analysis of T cell immunity in SARS-CoV-2 infection.

## Data Availability

Not applicable.

## Abbreviations

COVID-19: Coronavirus disease 2019
SARS-CoV-2: Severe acute respiratory syndrome coronavirus 2
CTL: Cytotoxic T cell
CRS: Cytokine release syndrome
IL: Interleukin
TNF: Tumor necrosis factor
IFN: Interferon
ACE2: Angiotensin-converting enzyme 2
MIS-C: Multisystem inflammatory syndrome in children
ILC: Innate lymphoid cells
TCR: T cell receptor
TRM: Tissue-resident memory
TEM: Effector memory T cell
TEMRA: Terminal differentiated effector T cell
TCM: Central memory T cell
TSCM: Stem cell memory T cell
HLA: Human leukocyte antigen
NSP: Non-structural proteins
UHD: Unexposed healthy donors
BM: Bone marrow
LN: Lymph node
PBMC: Peripheral blood mononuclear cells
pMHCI: Peptide-loaded major histocompatibility complex class I
VOC: Variants of concern
